# Clinical effect and prognostic factors of mechanical thrombectomy in the treatment of acute ischemic stroke

**DOI:** 10.12669/pjms.38.5.5723

**Published:** 2022

**Authors:** Liang Li, Peipei Cheng, Jiwei Zhang, Guang Wang, Tiemin Hu, Fan Sun

**Affiliations:** 1 Liang Li, Department of Neurosurgery, The Affiliated Hospital of Chengde Medical College, Chengde 067000, Hebei Province, P.R. China; 2 Peipei Cheng Department of Pediatrics, The Affiliated Hospital of Chengde Medical College, Chengde 067000, Hebei Province, P.R. China; 3 Jiwei Zhang, Department of Neurosurgery, The Affiliated Hospital of Chengde Medical College, Chengde 067000, Hebei Province, P.R. China; 4 Guang Wang, Department of Neurosurgery, The Affiliated Hospital of Chengde Medical College, Chengde 067000, Hebei Province, P.R. China; 5 Tiemin Hu, Department of Neurosurgery, The Affiliated Hospital of Chengde Medical College, Chengde 067000, Hebei Province, P.R. China; 6 Fan Sun, Department is Neurology. The Affiliated Hospital of Chengde Medical College, Chengde 067000, Hebei Province, P.R. China

**Keywords:** Acute ischemic stroke, Mechanical bolt removal, treatment, Clinical efficacy, Prognostic factors

## Abstract

**Objectives:**

To explore the clinical effect and prognostic factors of mechanical thrombectomy in the treatment of acute ischemic stroke.

**Methods:**

The records of patients with acute ischemic stroke treated in our hospital from April 2020 to April 2021 were retrospectively selected. A total of 65 patients were treated with mechanical thrombectomy. After treatment, they were scored with modified Rankin Scale (MRS). The treatment effect and prognostic factors were analyzed.

**Results:**

The occluded vessels were successfully opened in 65 patients. The recanalization rate was 96.92%. There were no serious complications of thrombectomy. The time from femoral artery puncture to vascular recanalization was (84.06±16.64) minutes and the number of thrombectomies was (2.52±0.71). There were 42 patients with good prognosis and 23 patients with poor prognosis. Analysis of the prognostic factors showed that the time from onset to admission in the good prognosis group was shorter, the NIHSS score before thrombectomy was higher, and the Alberta stroke program early CT Score (ASPECT) score was lower as compared to the patients in the poor prognosis group. The grade of vascular recanalization in the good prognosis group was better than that in the poor prognosis group, and the level of PCT was lower (P<0.05). Logistic regression analysis showed that the time from onset to admission, NIHSS and ASPECT scores before thrombectomy were the prognostic factors of mechanical thrombectomy in the treatment of acute ischemic stroke.

**Conclusion:**

Mechanical thrombectomy is effective in the treatment of acute ischemic stroke and can effectively promote the recanalization of occluded vessels, but the NIHSS and ASPECT scores from the onset to the time of admission before thrombectomy can directly affect the prognosis of patients.

## INTRODUCTION

Acute ischemic stroke is a common cerebrovascular disease with high mortality and disability rates. About 75.0% of the surviving patients have varying degrees of disability, which seriously affects their quality of life.^1^,^2^ At present, intravenous thrombolysis and mechanical thrombectomy are mostly used to treat acute ischemic stroke. Although intravenous thrombolysis has good curative effect and can effectively recanalize the occluded vessels, it has extremely strict requirements for the time window. The rate of intravenous thrombolysis in China is only 1.6% ~ 1.9%. Even if intravenous thrombolysis is successfully performed, the disability rate within 90 days is still as high as 60.0% ~ 80.0%.^3^

Mechanical thrombectomy can directly remove the thrombus at the occluded vessel, successfully recanalize the occluded vessel and improve the prognosis of patients. Studies show that in the treatment of acute ischemic stroke, compared with simple intravenous thrombolysis and conservative medical treatment, mechanical thrombectomy may result in 66.0%~88.0% recanalization rate of occluded vessels, and effectively reduce the disability rate of this disease.^4^ Acute ischemic stroke is characterized by acute onset and the treatment time window is short. Even if the treatment time window can be prolonged by six to eight hours after mechanical thrombectomy, the time from onset to femoral artery puncture will be delayed by one minute, which may cause a significant loss of thrombectomy benefits and increase the degree of disability in patients after the treatment.^5^ In recent years, treatment protocols for acute ischemic stroke have been continuously improved, leading to the improvement of the treatment success rate to a certain extent. However, in the actual clinical treatment process, there is marked variability in the clinical benefits for different patients receiving mechanical thrombectomy. It is very important, therefore, to evaluate the treatment of acute ischemic stroke by mechanical thrombectomy. A comprehensive analysis of prognostic factors can provide a reliable basis for the selection of treatment schemes and for evaluation of prognosis.^6^ In recent years, our hospital has mostly selected mechanical thrombectomy to treat acute ischemic stroke.

The aim of this study was to evaluate the efficiency of mechanical thrombectomy in promoting recanalization of occluded vessels in the treatment of acute ischemic stroke, and the impact of ASPECT score and other factors on the prognosis of patients.

## METHODS

Medical records of patients with acute ischemic stroke treated in our hospital from April 2020 to April 2021 were selected. Out of 65 patients, 35 were males and 30 were females.

### Inclusion criteria:

- Meet the diagnostic criteria in the guidelines for early management of patients with acute ischemic stroke^7^ issued by the American Heart Association/American Stroke Association in 2018;
- The modified Rankin Scale (MRS) score before onset was≤1;
- Onset time<24 hour;
- Complete clinical data available;
- Patients aware of the study and cooperate with informed consent.

### Exclusion criteria:

- Bleeding or bleeding tendency;
- Severe organ dysfunction or failure;
- Other intracranial diseases;
- There was uncontrollable hypertension before treatment;
- Incomplete medical records.

This study has been approved by the medical ethics committee of our college (approval number: CYFYLL2021103, date: 2018-Nov-13).

For the mechanical thrombectomy, patients were in the supine position, ECG monitoring, disinfection, towel laying and local anesthesia were carried out. 18G puncture was carried out for the left or right femoral artery by Seldinger wire technique, and 6F arterial sheath (11 cm) was inserted through guide wire. Cerebral angiography was done through 5F single curved catheter to evaluate the specific situation of occluded vessels. Under the guidance of Synchro-14 micro guide wire, angiography was performed on the distal end through Rebar-18 micro catheter across the occluded segment, the branches of occluded arteries were observed, and then the guide wire was withdrawn. The stent (4×20mm Solitaire) was crossed over the thrombus segment through the microcatheter, and released according to the length of the thrombus segment. Angiography showed that after the stent was in place, it was withdrawn slowly, and about 20ml of blood in the catheter was withdrawn after success. Angiography was performed again to observe the opening of the artery, and angiographic intracranial flow was evaluated by Thrombosis In Cerebral Ischemia (TICI) score^8^, with Grade≥2B indicating successful recanalization of the blood vessel. For those with grade<2B, stent thrombectomy was performed again. After operation, CT examination was performed immediately to evaluate the intracranial condition. Those with fresh bleeding were transferred to ICU, and those without fresh bleeding were transferred to general ward.

### Observation Index: Vascular recanalization, neurological function and prognosis:

Vascular recanalization was evaluated by TICI^9^,^10^: grade 0 - no blood perfusion at the distal end of occluded vessels; Grade-1- a small amount of blood perfusion can be seen in the distal branches of occluded vessels; Grade 2A- blood flow perfusion in the distribution area of occluded artery≤50.0%; grade 2B- blood flow perfusion in the distribution area of occluded artery>50.0%; Grade-3- complete blood perfusion can be seen in the distribution area of occluded artery. Grade≥2B indicated successful recanalization. The prognosis was evaluated by MRS score^11^: 0 for asymptomatic; one point for mild symptoms but no obvious disability; two points for mild disability but able to handle personal affairs; Moderately disabled, requiring some assistance,three points; Severe disability, walking and physical care need assistance,four points; Severely disabled and bedridden, five points; Death, six points. From onset to admission, vascular recanalization, National Institute of Health Stroke Scale(NIHSS) score before thrombectomy, Alberta stroke program early CT Score(ASPECT) score before thrombectomy, occlusion site, collateral circulation, thrombectomy times, procalcitonin(PCT) Leukocyte count^12^, hemoglobin level, for statistically significant factors, logistic regression analysis was carried out. Among them, NIHSS ranges from 0~45 points. The higher the score, the higher the degree of neurological deficit^13^; ASPECT scores ranged from 0~14. The lower the score, the more severe the degree of ischemia.^14^

### Statistical Analysis:

The data of this study were processed by spss22.0, [n(%)] represents the counting data. The test method is *x*^2^, (x̄ ± s) represents the measurement data. Normal distribution was tested by t-test, □=0.05 was the test level, the non-normal distribution was tested by rank sum test, and the risk factors were analyzed by logistic regression. P<0.05 was considered statistically significant.

## RESULTS

A total of 65 patients met the inclusion criteria, 35 males and 30 females. The mean age was (63.09±8.99) years. The patency rate of occluded vessels was 96.92% (63/65). The TICI classification showed that two cases were grade 2A (3.08%),18 cases were grade 2B (27.69%), and 45 cases were Grade-3 (70.77%). There were no serious complications of thrombectomy. The time from femoral artery puncture to vascular recanalization was (84.68±23.45) minutes, and the number of thrombectomy times was (2.12±0.46). Follow up to three months after operation showed that the MRS scores of 23 cases were 3~6 and of 42 cases- 0~2.

Based on the MRS scores after treatment, patients were divided into poor prognosis group and good prognosis group. there was no statistical difference in general information between the two groups (P>0.05), Table-I. By comparing and analyzing the prognostic factors of the two groups, it was found that the time from onset to admission in the good prognosis group was shorter than that in the poor prognosis group (P<0.05), the NIHSS score before thrombectomy was lower than that in the poor prognosis group (P<0.05), the ASPECT score was higher than that in the poor prognosis group (P<0.05), the vascular recanalization grade was better than that in the poor prognosis group (P<0.05), and the PCT level was lower than that in the poor prognosis group (P<0.05). There was no significant difference in occlusion site, collateral circulation, thrombus removal times, PCT, leukocyte count and hemoglobin level between the two groups (P>0.05) (Table-II). Logistic regression analysis was carried out for time from onset to admission, NIHSS before thrombectomy, ASPECT score, vascular recanalization grade and PCT level. It was found that the time from onset to admission, NIHSS before thrombectomy and ASPECT score were the prognostic factors of mechanical thrombectomy in the treatment of acute ischemic stroke (Table-III).

**Table I:** Comparison of general information between the two groups.

Group	n	Sex ratio (M/F)	Age (year)	Occlusion of blood vessels (n)		Causes of stroke (n)		

				Pre-circulation	Posterior circulation	Aorta	Mezzanine	Psychogenic
Control group	23	13/10	61.22±10.59	18	5	15	1	7
Observation group	41	22/20	64.12±7.94	34	8	30	3	9
*x*^2^/*t*	-	0.103	1.149	0.067		0.761		
*P*	-	0.749	0.258	0.795		0.683		

**Table II:** Comparison of prognostic factors between the two groups.

Factor		Control group (n=21)	Observation group (n=44)	*x*^2^/*t*	*P*
Time from onset to admission (h)		9.52±3.10	5.40±3.97	4.296	<0.001
Recanalization of blood vessels (n)	Class 2b	8	10	5.129	0.077
	Class3	13	32		
NIHSS score before thrombus removal (Score)		21.22±5.35	18.71±4.15	2.095	0.04
ASPECT score before thrombus removal (Score)		9.13±2.56	11.78±3.00	3.585	0.001
Occlusion of blood vessels (n)	Pre-circulation	18	34	0.103	0.749
	Posterior circulation	5	8		
Number of bolt removal(times)		2.43±0.66	2.57±0.74	0.740	0.462
PCT (ng/ml)		0.28±0.04	0.26±0.01	2.183	0.038
White blood cell count (×10^9^/L)		11.56±3.08	10.76±3.12	0.986	0.328
Hemoglobin (g/L)		14.69±2.99	13.95±3.04	0.947	0.347

**Table III:** Logistic regression analysis results.

Index	B	*S.E.*	*Wald*	*P*	*OR*	95% *CI*
Time from onset to admission	0.865	0.311	7.745	1	0.005	1.29~4.37
NIHSS score before thrombus removal	0.966	0.381	6.42	1	0.011	1.24~5.54
ASPECT score before thrombus removal	-0.278	0.134	4.301	1	0.038	0.58~0.98
PCT (ng/ml)	-56.807	29.395	3.735	1	0.053	0~2.24

## DISCUSSION

In this study, 65 patients with acute ischemic stroke were treated with mechanical thrombectomy. Our results showed that the occluded vessels of 65 patients were successfully opened, the vascular recanalization rate was 96.92%, and there were no serious thrombectomy complications. The time from femoral artery puncture to vascular recanalization was (84.06±16.64) minutes, and the number of thrombectomies was (2.52±0.71), suggesting that in the treatment of acute ischemic stroke, mechanical thrombectomy has good effect and can effectively recanalize the occluded blood vessels. Edgar A Samaniego et al.^15^ conducted a literature review study to describe early treatment options, such as venous tissue plasminogen activator and the latest mechanical thrombectomy (MT) technology. The recanalization rate with new technology and MT equipment was close to 90%. Timely interventions also had better clinical results, and about 50% of patients achieved functional independence within 90 days. Serge Bracard^16^ in a multi-center clinical controlled trial involving 404 patients also showed that mechanical thrombectomy combined with standard intravenous thrombolysis can improve the prognosis and functional independence of patients with acute cerebral ischemia. Mechanical thrombectomy has gradually become a new treatment scheme for intracranial large artery occlusion. Compared with intravenous thrombolysis, mechanical thrombectomy has a higher recanalization rate, can prolong the treatment time window, thus effectively improving the prognosis of patients.^17^

Pathological process of acute ischemic stroke is mainly acute occlusion of cerebral vessels. This leads to the interruption of blood flow supply to brain tissue, and, subsequently, to ischemic and hypoxic brain injury, neuronal damage, and disability.^18^ Liet W al.^19^, in a study evaluating the prognosis of re-occlusion after mechanical thrombectomy (MT) in Chinese stroke patients, found that NIHSS score, 90 day MRS score (4:3) and 90 day mortality in the re-closure group were higher than those in the non-re-closure group. The results of our study focused on several prognostic factors of acute thrombectomy in the treatment of acute ischemic stroke, such as time from onset to admission, NIHSS score before thrombectomy and ASPECT score. Brain tissue is highly sensitive to ischemia and hypoxia, and the time of cerebral vascular occlusion directly correlates with the time of cerebral ischemia and hypoxia and the degree of neuronal injury and poor prognosis. Studies showed that mechanical thrombectomy can prolong the time window of anterior circulation large vessel occlusion treatment to six to eight hour after the onset of symptoms, but within the treatment time window, the prognosis of patients is still closely related to the start time of treatment. For every 30 minutes extension of vascular recanalization time, the probability of good prognosis within 90 days will be reduced by 12.0%.^20^ NIHSS score is a common index to reflect the degree of neurological deficit. The higher NIHSS score, the larger the core infarct area and the more serious the degree of neurological deficit. Studies show that the NIHSS score of acute ischemic stroke patients with poor prognosis is significantly lower than that of patients with good prognosis, which is consistent with the results of this study.^21^ ASPECT score can reflect the core infarct area. The lower the score, the larger the infarct area and the worse the prognosis. The results of our study show that when mechanical thrombectomy is used to treat acute ischemic stroke, the prognosis of patients is directly affected by the time from onset to admission, and NIHSS and ASPECT scores before thrombectomy. Our results have clear clinical implications for developing and optimizing clinical treatment scheme of acute ischemic stroke. This will allow patients to get effective timely treatment after onset, shorten the time of cerebral ischemia and hypoxia, reduce the degree of neurological deficit, reduce the core infarct area, and, therefore, improve the effectiveness of mechanical thrombectomy and overall prognosis.

### Limitations of the study:

It includes a small sample size (65 patients), few observation indicators and no long-term follow-up, which may make the conclusion one-sided and limited. Therefore, further studies with larger sample sizes, observation indexes and extended follow-up times are needed to objectively analyze the clinical effect and prognostic factors of mechanical thrombectomy in the treatment of acute ischemic stroke.

## CONCLUSION

In the treatment of acute ischemic stroke, mechanical thrombectomy is effective in quick re-opening of occluded vessels. However, the prognosis of patients is affected by the time from onset to admission, NIHSS and ASPECTS scores before thrombectomy.

### Authors’ contributions:

**LL, TH & FS:** Conceived and designed the study.

**PC, JZ, GW & FS:** Collected the data and performed the analysis.

**LL** **& PC:** Were involved in the writing of the manuscript and are responsible for the integrity of the study.

All authors have read and approved the final manuscript.
